# Analysis of spatial heterogeneity in normal epithelium and preneoplastic alterations in mouse prostate tumor models

**DOI:** 10.1038/srep44831

**Published:** 2017-03-20

**Authors:** Mira Valkonen, Pekka Ruusuvuori, Kimmo Kartasalo, Matti Nykter, Tapio Visakorpi, Leena Latonen

**Affiliations:** 1 Prostate Cancer Research Center, Faculty of Medicine and Life Sciences and BioMediTech, University of Tampere, Tampere, Finland; 2 Tampere University of Technology, Pori, Finland; 3 BioMediTech Institute and Faculty of Biomedical Sciences and Engineering, Tampere University of Technology, Tampere, Finland; 4 Fimlab Laboratories, Tampere University Hospital, Tampere, Finland

## Abstract

Cancer involves histological changes in tissue, which is of primary importance in pathological diagnosis and research. Automated histological analysis requires ability to computationally separate pathological alterations from normal tissue with all its variables. On the other hand, understanding connections between genetic alterations and histological attributes requires development of enhanced analysis methods suitable also for small sample sizes. Here, we set out to develop computational methods for early detection and distinction of prostate cancer-related pathological alterations. We use analysis of features from HE stained histological images of normal mouse prostate epithelium, distinguishing the descriptors for variability between ventral, lateral, and dorsal lobes. In addition, we use two common prostate cancer models, Hi-Myc and *Pten*+/− mice, to build a feature-based machine learning model separating the early pathological lesions provoked by these genetic alterations. This work offers a set of computational methods for separation of early neoplastic lesions in the prostates of model mice, and provides proof-of-principle for linking specific tumor genotypes to quantitative histological characteristics. The results obtained show that separation between different spatial locations within the organ, as well as classification between histologies linked to different genetic backgrounds, can be performed with very high specificity and sensitivity.

Tissue histology is one of the main determinants in studying and diagnosing many pathologies, including cancer. Solid tumors change the structure of the tissue due to altered morphologies and localizations of tumor cells within the normal tissue. Histopathology is traditionally a very intuitive method, where decisions are most often based on visual inspection. Often, however, ability to objectively recognize and quantify pathological changes in tissue histology would be desired. Furthermore, gaining the decisive pathological information from basic histological stainings, most often hematoxylin and eosin (HE), would help to avoid using costly special stainings. Several current approaches aim to develop tools to help clinical pathologists to determine presence or state of a particular lesion from HE-stained images, e.g. to stage cancer for diagnostic and prognostic purposes^1^,^2^. Yet, a pressing need to diagnose cancer at earlier stages concerns several cancer types, e.g. prostate cancer and breast cancer; when tumors are still small and changes in them more benign, treatment options and prognoses are better. Ability to recognize and quantify small and subtle changes in tissue morphology are crucial also to basic and preclinical research aiming to identify early changes preceding and leading to malignant stages of cancer.

To be able to quantify changes in tissue morphology, measurable determinants of the pathology in question need to be determined. Key questions are how to differentiate between normal and pathological tissue, how to measure the stage of the pathological change, and even how to differentiate between several possible types of change, e.g. subtypes of cancer. For example, accurate separation of pathologies from normal tissue histology requires understanding and inclusion of all states and variables of the normal tissue, whether originating from characteristics of the tissue itself, or technical variation due to e.g. orientation of the histological section cut relative to tissue structures.

In cancer research, recent years of next-generation sequencing have revealed the extent of genetic and gene expression alterations in cancer^3^,^4^. However, the phenotypic effects of many genetic alterations and their combinations is still under research. The common goal is to be able to subtype tumors better for enhanced patient stratification and care in the future. Combination of genetic information with histology, however, requires that the histological information can be transformed into a quantitative, objective form. This can be achieved through digital imaging and computational analysis of the histological characteristics. While computational image informatics can provide a plethora of quantified descriptors of a given image, the challenge in histology is to sort out the relevant characteristics which can be presented in the form of useful feature representations.

Feature-based analysis combined with supervised learning has been a common approach in decision support systems and computer aided diagnosis based on whole slide images^1^,^5^,^6^,. Such approaches have been successfully used for quantitatively describing characteristics of prostate histology in neoplastic lesions both for a mouse model^7^ and for human tissue^8^. However, previous studies have left room for improved feature engineering and classification performance.

We aim at improving histological recognition and quantification of pathological features in prostate cancer, and search for descriptors to differentiate early pathological lesions from normal prostate tissue. Furthermore, the goal is to separate genetically different types of early neoplastic changes from each other. In here, we use two classical and popular genetic prostate cancer mouse models, namely heterozygous *Pten*^9^ and Hi-Myc^10^, to perform quantitative image analysis on early neoplasms compared to normal mouse prostatic tissue. With a computational separation of hundreds of features from the whole slide images of histological tissue sections and a random forest based machine learning approach, we find a combination of tissue features able to distinguish between 1) normal spatial heterogeneity in the prostate tissue, 2) early *Pten*+/− or Hi-Myc-induced neoplasms from normal tissue, and 3) the two types of neoplasms from each other. Our study serves as the first step towards developing tools for automated analysis of early neoplastic changes in prostate tissue and their linkage to different genetic groups.

## Results

### Spatial variation in epithelium of normal prostate

**Figure 1:**
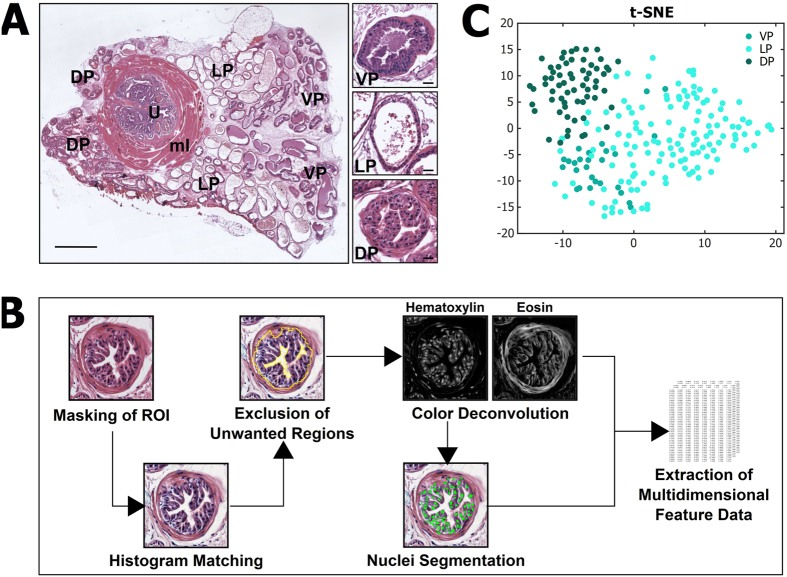
Quantitative image analysis of normal mouse prostate histology. (**A**) General appearance of mouse prostate in a histological section (large image) and variation in the appearance of normal epithelium within three lobular areas of mouse core prostate: ventral prostate (VP), lateral prostate (LP) and dorsal prostate (DP) (small images). Urethra (U) and the muscle layer (ml) surrounding it are marked. Scale bars: 1 mm (large image) and 25 *μ*m (small images). (**B**) Overview of the image analysis workflow that includes masking of ROI, correcting color variation using histogram matching, excluding areas not including tissue, separating hematoxylin and eosin stains, segmenting cell nuclei, and extracting quantitative feature data. (**C**) A t-SNE plot presenting the spatial variation of quantitative features in the epithelium between the normal mouse prostate lobes (VP, LP, DP).

First, we wanted to assess normal spatial variation in the histology of the prostate. Mouse core prostate can be divided in three lobular areas: ventral prostate (VP), lateral prostate (LP) and dorsal prostate (DP), which surround the urethra (Fig. 1A). All three lobes are dominated by prostate acini covered with an epithelial cell layer and are surrounded by a basement membrane and loose connective tissue. Between the lobes, subtle differences exist in the organization and direction of the acinar tubes, somewhat affecting the appearance and lumen size of acini in histological preparations. The epithelium is of specific relevance due to being the tissue component where prostate cancers originate from. The normal appearance of the epithelium varies between the different lobes (Fig. 1A). Epithelium in the DP is columnar, cytoplasm is relatively eosinophilic, and the nuclei are centrally to basally located. The epithelium can be tufted. LP epithelium has only sparse infoldings. The cells are cuboidal or low columnar, and the nuclei are small, uniform and basally located. VP has only focal epithelial tufting. The cells are cuboidal to columar, and the cytoplasm is less eosinophilic. Nuclei are small and basally located (Fig. 1A).

We manually selected 227 acini to represent variation in prostate epithelium in histological sections, including both the heterogeneity in normal appearance of the tissue, and the technical variance (e.g. acini cut in different orientations). For these images, we performed preprocessing to, for example, correct color variation across the tissue samples, and to exclude areas not including tissue (e.g. empty and secretion-containg areas inside the acini) (Fig. 1B). We applied color deconvolution to separate hematoxylin and eosin stains as separate color channels, and performed nuclear segmentation. The resulting image information we used to compute a compilation of 241 features (listed in Supplementary Table 1). These features included numerous descriptors related to tissue texture and local environments, as well as numeric representations of properties, spatial arrangement, and distribution of nuclei. When these 241 features are used to represent the samples in dimension-reducing t-Distributed Stochastic Neighbor Embedding (t-SNE)^11^ plot, the extent of the extractable spatial variation between the normal lobes of the prostate is shown (Fig. 1C). While the VP shares characteristics within the range of LP, the DP is more clearly distinguished from the other lobes.

### Quantitative characteristics of mPIN lesions

**Figure 2:**
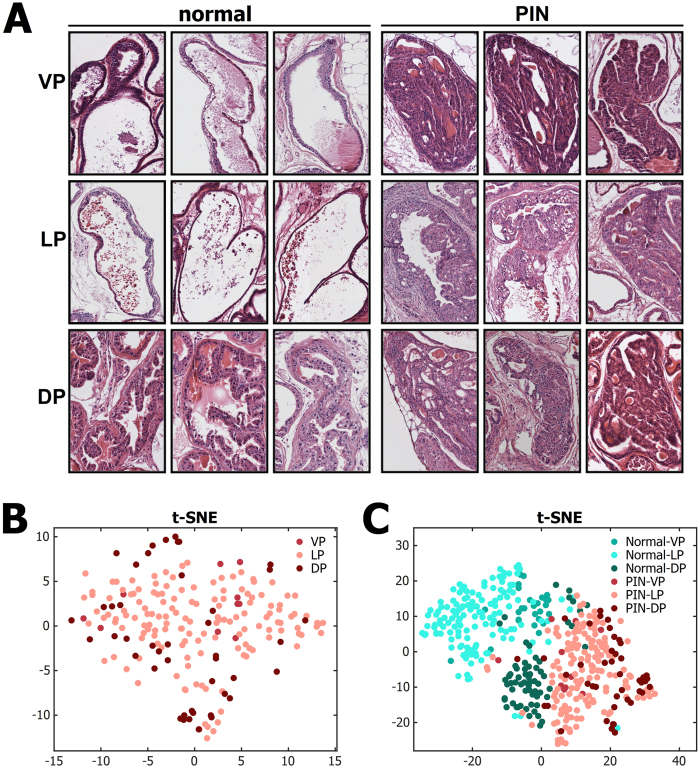
Quantitative characteristics of mouse PIN. (**A**) Examples of prostatic acini containing normal epithelium and PIN lesions from the three lobular areas (VP, LP, DP). (**B**) Representation of the quantitative features of PIN lesions in three mouse prostate lobes (VP, LP, DP) in two-dimensional feature space using t-SNE. (**C**) A t-SNE visualisation reveals decreasing spatial heterogeneity in PIN lesions compared to normal epithelium in the mouse prostate.

To study distinction of small pathological changes from normal epithelium, we wanted to compare normal tissue to early pathological lesions. Mice heterozygous for tumor suppressor *Pten* form mouse prostatic intraepithelial neoplasia (mPIN) within 8–12 months^9^. In here, we used prostate samples from *Pten*+/− mice of 10-11 months, when recognizable mPIN is evident (Fig. 2A).

We selected 199 areas of mPIN, and performed image processing and feature computation as above. A t-SNE plot (Fig. 2B) shows that the PIN lesions of different prostate lobes are mixed rather than separated as lobe-specific clusters. This indicates that, compared to normal epithelium, the spatial heterogeneity is decreased in mPIN (compare Fig. 2B to Fig. 1C). When comparing the relative distributions of normal epithelium and mPIN lesions, PIN areas are clearly separate from normal tissue areas by the computed compilation of features (Fig. 2C). When comparing the different prostatic lobes, it is evident that LP is furthest and DP closest to PIN lesions based on the computed feature profile, corresponding to the tissue characteristics observed by eye (Fig. 2A).

**Figure 3:**
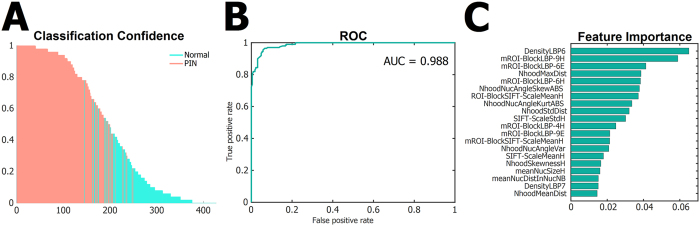
Classification of mouse PIN using supervised machine learning. (**A**) Classification confidence given by the random forest model for each *Pten*+/− sample to belong to the group of PIN using LOOCV. (**B**) The sensitivity and specifity of the random forest model classification shown in A presented as a ROC curve, and the performance measured by area under the curve (AUC). (**C**) List of 20 most significant features and their relative importances in the separation of mPIN from normal epithelium given by a classification model trained with all *Pten*+/− samples.

To test whether the computed feature characteristics can be used to reliably separate mPIN lesions from normal epithelium, we applied machine learning. We developed a random forest based model and applied it in leave-one-out cross validation (LOOCV) to estimate the probability of a sample to belong to the group of PIN lesions based on the feature data. Figure 3A shows the classification confidence given by the machine learning model for each sample to belong to the group of PIN. The accuracy of the estimations by the model was analysed using receiver operating characteristic (ROC) curve, from which the area under curve (AUC) measure can be used for quantifying the separation between the normal and preneoplastic tissues (Fig. 3B; *AUC* 0.988). Predictor importances of 20 most influential features in the model to distinquish between the morphology types are shown in Fig. 3C. These feature importances were given by a model that was trained with all available samples.

From the LOOCV experiment, used to validate the robustness of our random forest model, we collected altogether 426 models from which the average importances are shown in Supplementary Figure 1. The averaged feature importance list contains a similar set of features as that given by the original model trained with all available samples (Fig. 3C). These include several types of texture features, such as LBPs and SIFT-features. Nuclear features include several descriptors of nuclear size, density and neighbourhood (NhoodMaxDist, NhoodStdDist, HhoodSkewness, meanNucSize, meanNucDistInNucNB, NhoodMeanDist). Another set of important features are the features describing the relative positions and orientations of the nuclei (NhoodNucAngleSkewABS, NhoodNucAngleKurtABS, NhoodNucAngleVar, NhoodNucAngleStd0). These capture the distinctive property of normal epithelial tissue, where nuclei are most often oriented as “beads in a row” as opposed to scattered distribution in a tumor (Supplemental Fig. 2).

We further tested the ability of the model to distinguish mPIN from normal epithelium in each lobe (Supplementary Figure 3). According to the different incidence of mPIN in the prostatic lobes in the *Pten*+/− model mice, the number of mPIN samples in the analysis varied between lobes being greatest in LP and lowest in VP (normal epithelium and mPIN *n*_*VP*_ = 37, *n*_*LP*_ = 282, *n*_*DP*_ = 107). PIN was distinguished from normal prostate most accurately in LP (*AUC* 0.997), likely due to the clear phenotypic difference in the histology. mPIN in VP and DP were similarly challenging, although with these, relatively small sample sets, a very high accuracy was still reached (*AUC* 0.972).

### Computational distinction between histologies of different genetic groups

**Figure 4:**
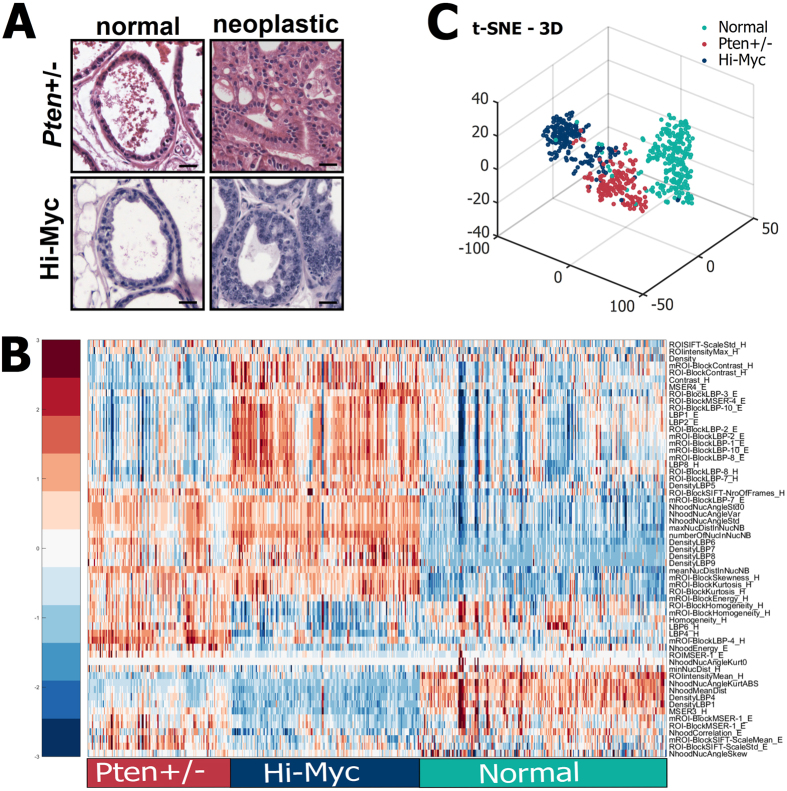
Quantitative characteristics of normal epithelium, *Pten* heterozygous mPIN, and Hi-Myc-induced preneoplasia in mouse lateral prostate. (**A**) Examples of representative histologies of normal and preneoplastic LP epithelium from *Pten*+/− and Hi-Myc mouse model prostates. Scale bars 25 *μ*m. (**B**) Distinct feature value patterns of *Pten* heterozygous PIN, Hi-Myc-induced early neoplasia, and normal epithelium presented in a heatmap after hierarchical clustering of normalized feature values. (**C**) Three-dimensional t-SNE visualisation shows distinctive patterns for the three histological populations.

High expression of oncogenic Myc in the mouse prostate induces neoplastic lesions visible already at 1 month of age^10^. These lesions develop later on to adenocarcinoma, in contrast to the mPIN in the *Pten*+/− heterozygous mice which does not develop into carcinoma without additional genetic or carcinogenic manipulation^9^. We wanted to compare these two types of early neoplasms with genetic differences, and to find computational features separating them from each other and from the normal epithelium. As most of the tumors in these models form in the LP, we concentrated on this tissue area selecting only LP samples from *Pten*+/− model mice (normal epitheliu *n* = 137, mPIN *n* = 145), and manually selected samples from LP of Hi-Myc mice (normal epithelium *n* = 111, mPIN *n* = 189). Examples of representative histologies on normal and preneoplastic LP epithelium are shown in Fig. 4A.

As the two preneoplastic change types occur at different ages, our samples from the two different models represented prostate epithelium from mice of very different age (10–11 months in *Pten*+/− mice compared to 1 month in Hi-Myc mice). Thus, to ensure consistency and comparability of the data from hiMyc and *Pten*+/− tissues, we selected features whose distributions did not show statistically significant difference (threshold *α* = 0.05) according to Kolmogorov-Smirnov test in the two control groups to use in the further analysis. Altogether 59 features fulfilled the criterion (Supplementary Table 2, Supplementary Figure 4). It is evident already by the feature values that the three groups of samples have their own, distinct signatures (Fig. 4B). Furthermore, these groups are clearly distinguished in representation of the samples according to the feature values in t-SNE (Fig. 4B).

**Figure 5:**
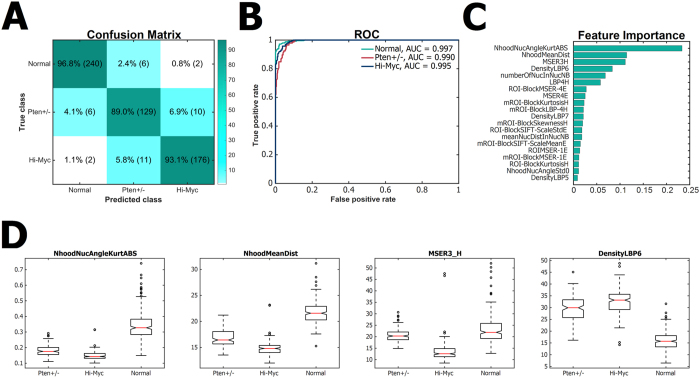
Classification between normal epithelium, *Pten* heterozygous mPIN, and Hi-Myc-induced preneoplasia in mouse lateral prostate using supervised machine learning. (**A**) Classification accuracies for separating the three histological populations given by the proposed classification model as a confusion matrix. (**B**) ROC curves representing the performance of the classification model when distinguishing *Pten* heterozygous mPIN, Hi-Myc-induced early neoplasia, and normal epithelium. Each curve presents the classification accuracy for separating one group of the three from the two of the other groups. Performances as measured by the area under the curve (AUC) are shown. (**C**) List of 20 most significant features and their relative importances in the separation of *Pten* heterozygous mPIN, Hi-Myc-induced early neoplasia, and normal epithelium by the random forest model trained with all of the LP samples from *Pten*+/−, Hi-Myc, and normal epithelium. (**D**) Values of four most significant features in C as boxplots showing the differences between *Pten* heterozygous mPIN, Hi-Myc-induced early neoplasia, and normal epithelium.

We developed a machine learning model based on the selected subset of features to estimate the probability of a sample to belong to any of the three groups (normal epithelium, *Pten* heterozygous mPIN, or Hi-Myc-induced early neoplasia). The model is successful in predicting accurately the histological classes of the samples, as shown in a confusion matrix of the predictions (Fig. 5A) and a ROC-curve of accuracy analysis (Fig. 5B; *AUC* 0.997 for normal, 0.990 for *Pten*+/−, and 0.995 for Hi-Myc). Similarly as in distinguishing mPIN from normal epithelium, the predictor importances of the most valuable features in this random forest model (Fig. 5C) include nuclear density and angle-related features (NhoodNucAngleKurtABS; NhoodMeanDist, numberOfNucInNucNB). As expected, however, the separation of two morphologically different neoplastic histologies brings features capturing more detailed textural information from the stain intensities and from nuclear density map to the top list of features (e.g. DensityLBP6, LBP4H). These remain in the list of most influential features even when averaging the predictor importances of 582 trainings of the random forest model (Supplementary Figure 5), and provide detailed, quantitative information of the differences between Hi-Myc and *Pten*+/− -provoked early neoplasms (Fig. 5D).

## Discussion

Automatic recognition and quantitative analysis of tissue pathologies requires ways to computationally identify representative histological features separating normal and altered tissue. In this work, we extracted a set of 241 features from images of prostate tissue, and used them to analyze spatial heterogeneity within normal prostate, as well as to separate different types of early neoplastic changes from normal tissue and from each other. Our results show that separation between different spatial locations within the organ, as well as classification between histologies provoked by different genetic lesions, can be performed with very high specificity and sensitivity.

We applied traditional machine learning approach based on extraction of a large set of engineered features followed by a random forest ensemble classifier. Given the very high accuracy obtained for the relatively low number of samples and small size of regions of interest, this approach appears to be well justified. Recently, neural network based deep learning approaches^12^ have gained much attention in efforts to recognize cancerous tissue from normal tissue, and in developing pre-screening tools for pathologists to indicate suspect areas in tissue^13^,^14^. The set-back of these approaches so far is the difficulty of examining the relevance and meaning of model properties, i.e. network weights and output layer values used in the decision flow in the context of the tissue. In order to interpret the model properties used computationally to a pathologist or a research biologist, meaningful and recognizable features of the tissue are preferred, especially if the information of the tissue features need to be combined with readouts obtained from other measurement modalities. Methods to avoid the interpretability issues in using deep learning are likely to follow, while combination approaches of deep learning and traditional, feature based machine learning are also being investigated^15^,^16^.

In this study, we used two popular prostate cancer mouse models^17^, to compare the histology of early pathologies originating from different genetic alterations. Pten is a tumor suppressor that functions by inhibiting Akt pathway, and it is often deleted in human prostate cancer^18^. Mice heterozygous for *Pten* form cancer in many organs^9^. In the prostate, these mice develop mPIN^9^, which is a type of *in situ* lesion not reported to develop to invasive carcinoma without additional genetic or carcinogenic manipulation. Myc, on the other hand, is a transcription factor and a powerful oncogene overexpressed in many cancers, including prostate cancer^19^. Hi-Myc mice^10^ are a popular model to study prostate cancer, as it is one of the few genetic mouse models forming adenocarcinoma, thus exhibiting the cancer type most common in human prostate. Both our models represent an early phase in the step-wise development of cancer, and thus can be used in studying the early pathological changes in tissue. We achieved a successful separation between the histologies provoked in these two models by their different genetic alterations with very high specificity and sensitivity. This gives promise for future aims to automatically link genetic and quantitative histological information for more varied genetic populations and tumor subtyping.

The method we presented here is generic, and the applicability is not limited to mouse tissue. Tumor material from human patients, however, includes higher genetic and phenotypic variability compared to genetically restricted mouse model material. Thus, well annotated, large enough datasets are required to further develop and validate automated image analysis pipelines for future use in clinical and research applications in human cancer. Another current challenge is to automate the detection step of regions of interest. Recently, machine learning has been applied in automated ROI detection in, e.g., metastasis detection from human breast cancer samples^13^. Our approach for ROI classification could also be applied for ROI detection. This requires quantitative processing of not just the interest areas, but all the neighbouring tissue types and structures as well, including the basement membrane, stromal cells and fibers, nerves, vasculature, smooth muscle, and adipose tissue. Acquiring quantitative representations of these different tissue components and types will benefit applications of digital pathology also beyond cancer research.

In addition to tumor genotypes, data representing tumor phenotypes are desired to combine with quantitative representations of histology provided by methods such as the ones presented here. Recent advances in spatial transcriptomics^20^,^21^ integrated with computational histological analyses will undoubtedly provide understanding of spatial variation and evolution of tumors. Further, quantitative representation of tissue features and heterogeneity along with three-dimensional reconstructions of whole organs from serial sections, such as the prostate^22^, will enable intuitive visulization and provide novel insight into the spatial variation within tissue, as well as tumor growth patterns, in a natural context.

## Methods

### Ethical permissions

All animal experimentation and care procedures were carried out in accordance with guidelines and regulations of the national Animal Experiment Board of Finland, and were approved by the board of laboratory animal work of the State Provincial Offices of South Finland (licence numbers ESAVI/6271/04.10.03/2011 and ESAVI/5147/04.10.07/2015).

### Tissue samples

FVB/N mice either heterozygous for *Pten (Pten*+/−9) or transgenic for *MYC* oncogene (Hi-Myc^10^) were used. Prostates were fixed in PAXgene^TM^ tissue fixative according to manufacturer’s protocol, and embedded in paraffin. 5 *μ*m tissue sections were cut, attached to glass slides, and stained with hematoxylin and eosin.

### Imaging and ROI separation

HE-stained slides were whole slide imaged with Zeiss Axioskop40 microscope (Carl Zeiss MicroImaging, NY, USA) with 20x objective and a CCD color camera (QICAM Fast; QImaging, Canada) and a motorized specimen stage (Märzhäuser Wetzlar GmbH, Germany). The automated image acquisition was controlled by the Surveyor imaging system (Objective Imaging, UK). Uncompressed bitmap output was converted by JVSdicom Compressor application to JPEG2000 WSI format^23^. Snapshot images for Figures were obtained through JVSView virtual microscope (http://jvsmicroscope.uta.fi) and ImageJ software (National Institutes of Health, Bethesda, MD, USA^24^). Regions of interest were manually marked using a freehand selection tool in ImageJ. The resulting binary mask was used for extracting the ROI from the full resolution original HE image for further processing. In normal tissue, epithelial layer of prostate acini was included, excluding other tissue components in the organ such as stroma, urethra, vessels and nerve bundles. Pathological lesion masks each included solely mPIN/neoplastic epithelium. In the case of Hi-Myc samples, these were always within a single acinus each. In the case of *Pten*+/− lesions, where one mPIN tumor could reach several acinar lumen within a certain section, all affected lumen were included in a single mask.

### Preprocessing of images

The preprocessing of the images included histogram matching in order to remove the color variation between samples, exclusion of unwanted regions, color deconvolution to separate hematoxylin and eosin stains, and nuclei segmentation. These steps were implemented for bounding box areas around each ROI.

Histogram matching was performed to balance staining variation between sections. For a reference histogram, a mean histogram was computed from a representative set of samples consisting of ROI images of preneoplastic lesions and normal prostate epithelium from all three lobular areas. After this, histogram matching was performed by using a transform function computed between the image’s histogram and the reference histogram.

To segment the effective tissue area within each ROI, a mask for secretion-filled regions and empty areas was obtained by subtracting different color channels and performing contrast limited mapping of the intensity values similarly as in Ruusuvuori *et al*.^7^. The final binary mask was obtained by thresholding using Otsu’s method^25^. To smoothen the binary segmentation mask, morphological opening, closing, and filling were performed.

A color deconvolution algorithm^26^ was applied to convert the red, green, and blue channels of each image into hematoxylin stain, eosin stain, and background. Hematoxylin stains mainly the cell nuclei and therefore, hematoxylin channel was further processed to segment cell nuclei. Maximally stable extremal regions (MSER)^27^ were extracted from the grayscale image of hematoxylin stain. MSER is a method for blob detection from an image. Set of detected regions were selected based on the size corresponding to potential nuclei size. Additionally, regions that did not contain high grayscale intensity values related to high amount of hematoxylin stain, were excluded. To get the map for high rate of hematoxylin, tophat filtering, maximum filtering, Gaussian filtering, and image intensity adjustment was performed. Binary mask for high hematoxylin rate was obtained by thresholding using Otsu’s method^25^. To get the final binary mask for cell nuclei, MSER regions that were overlapping with mask for high rate of hematoxylin were selected.

### Feature extraction

Properties of each ROI were described with extraction of 241 features (Supplementary Table 1). These features included local descriptors related to image texture and distribution of nuclei. Texture features were extracted from local neighborhoods representing distinct levels of tissue architecture and, thus, measured as different features (e.g. Contrast-H, NhoodContrast-H, ROI-BlockContrast-H, ROIContrast-H), and also from both hematoxylin and eosin channels (e.g. Contrast-H, Contrast-E). The neighborhoods for texture features included bounding box of each segmented nucleus, 35 × 35 pixel neighborhood around each nucleus, non-overlapping 50 × 50 pixel neighborhoods within effective tissue area with unwanted regions included and excluded (e.g. mROI-BlockContrast-H, ROI-BlockContrast-H), and the whole bounding box image of the ROI. Nuclei distribution features were extracted from a 100 × 100 pixel neighborhood around each nucleus. To obtain a single feature vector for the whole ROI area, mean feature values were calculated from all blocks presenting one combination of certain feature and neighborhood. Details about each extracted feature and the applied local neighborhoods are presented in Supplementary Table 1.

### Texture features

The extracted texture based features included, e.g., mean intensity value, contrast, correlation, and energy, calculated from gray level co-occurrence matrix (GLCM). Properties of the texture within each ROI were also extracted using local binary patterns (LBP)^28^,^29^ and scale-invariant descriptors obtained via the Scale-invariant feature transform (SIFT)^30^. Additionally, properties of MSER regions were used as features. VLFeat^31^ implementations of SIFT and MSER were used in this work.

### Nuclei distribution features

Features related to distribution of cell nuclei were calculated from a nuclei location map, generated by marking the center point of each segmented nucleus. Features included descriptors related to inter-nuclei distance, nuclei locations with respect to each other described with angular statistics, number of nuclei within a neighborhood, and density features. The density features were calculated from a Gaussian filtered nuclei location map.

The angular statistic features were extracted using CircStat toolbox^32^. For each nucleus, an angle to all its neighbouring nuclei within a 100 × 100 pixel block was calculated. Supplementary Figure 2 presents an example polar histogram of these calculated angles from both normal epithelium sample and preneoplastic lesion sample. The features related to angular statistics included properties of this polar histogram, such as, variance, standard deviation, skewness, and kurtosis.

### Feature selection

To study the spatial variation in epithelium within different lobular areas of normal tissue, as well as in the comparison of normal tissue and preneoplastic mPIN lesions, we used all the extracted 241 features. For the comparison of three groups (normal, *Pten*+/−, and Hi-Myc), a feature selection was performed by statistical testing between features extracted from both Hi-Myc and *Pten*+/− normal prostate epithelium samples. Two-sample Kolmogorov-Smirnov test^33^ (significance threshold *α* = 0.05) was used to determine if the feature data extracted from these two normal sample groups were from the same continuous distribution. Altogether 59 features not showing statistically significant differece between the two normal populations were included in further analysis (Supplementary Table 2).

### Classification

The feature representations of ROI samples were used to train a random forest model^34^. Random forest algorithm was chosen due to its capability to handle both high data dimensionality and varying sample sizes in a computationally efficient manner. Additionally, the algorithm assigns weights for input features based on their importance in the classification task, providing an interpretable classification model and additional insight in the contribution of features. Bootstrap aggregation, which is a machine learning algorithm that combines multiple versions of decision trees into a random forest model, was used to improve the stability and accuracy of the model. Each decision tree version is constructed from a randomly sampled dataset with replacement. The implemented model was an ensemble of 50 decision trees.

Leave-one-out cross-validation was used for estimating the classification performance of or our random forest model. For example, when distinguishing mPIN from normal epithelium, we had 426 samples in total, and therefore, we trained 426 random forest models. For each model, one sample was left out from the training phase and then the trained model was used to predict the probability for this excluded sample to belong to the group with early neoplastic changes.

From the LOOCV experiments, average feature importances and corresponding standard deviations were compiled. When distinguishing mPIN from normal epithelium, these were calculated from the feature weights given by each of the trained 426 models (Supplementary Figure 1). When distinguishing between Hi-Myc-induced early neoplasia, *Pten* heterozygous mPIN, and normal epithelium, the average feature importances of the 582 models were calculated (Supplementary Figure 5).

## Additional Information

**How to cite this article:** Valkonen, M. *et al*. Analysis of spatial heterogeneity in normal epithelium and preneoplastic alterations in mouse prostate tumor models. *Sci. Rep.* **7**, 44831; doi: 10.1038/srep44831 (2017).

**Publisher's note:** Springer Nature remains neutral with regard to jurisdictional claims in published maps and institutional affiliations.

## Supplementary Material

Supplementary Figures and Tables
