# Supplementary material for: Analysis of spatial heterogeneity in normal epithelium and preneoplastic alterations in mouse prostate tumor models
Source: Sci Rep. 2017 Mar 20;7:44831. doi: 10.1038/srep44831 (PMC5357939; doi:10.1038/srep44831)
Supplement: Supplementary Figures and Tables [file srep44831-s1.pdf]

Analysis of spatial heterogeneity in normal epithelium and preneoplastic alterations in mouse prostate tumor models – Supplementary material

Mira Valkonen, Pekka Ruusuvuori, Kimmo Kartasalo, Matti Nykter, Tapio Visakorpi, Leena Latonen

Supplementary figures and tables

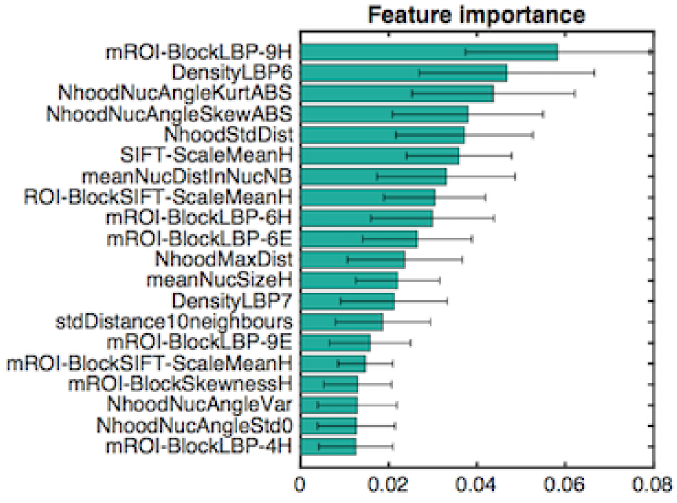

Supplementary Figure 1. The average feature importances and corresponding standard deviations compiled from 426 models trained to distinguish mPIN from normal epithelium in *Pten+/-* mouse prostate.

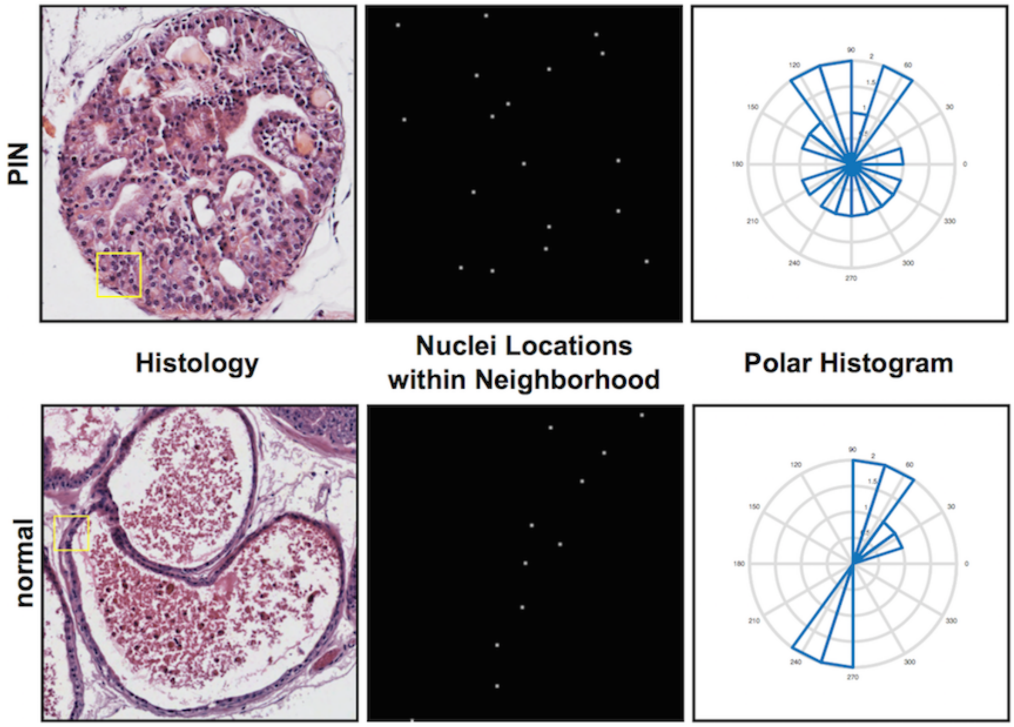

Supplementary Figure 2. Examples of nuclei distribution features that describe the arrangement of the nuclei. The figure presents 100x100 pixel neighborhood of nuclei locations extracted from samples of mPIN and normal in *Pten+/-* mouse prostate. The polar histogram presents the angles between the center nuclei and all other nuclei within the neighbourhood. Angular statistics extracted from this histogram are used as nuclei distribution features.

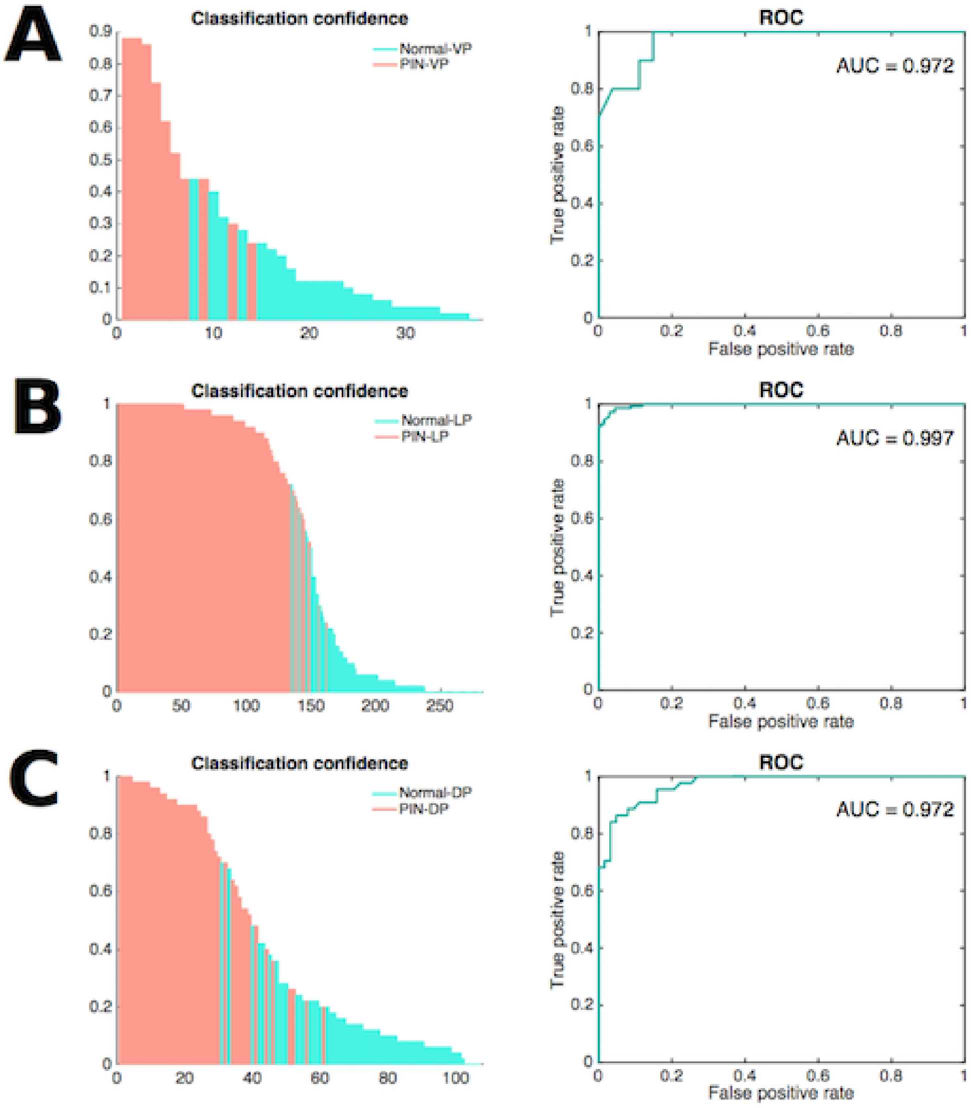

Supplementary Figure 3. The classification performance of the random forest machine learning model when distinguishing mPIN from normal epithelium in *Pten+/-* mouse prostate lobe-specifically. Results for each lobe are shown in (**A**) VP, (**B**) LP, and (**C**) DP. The figures on the left present the classification confidences given by the random forest models for each *Pten+/-* sample to belong to the group of PIN using LOOCV. The figures on the right present the corresponding ROC curves and calculated AUC values.

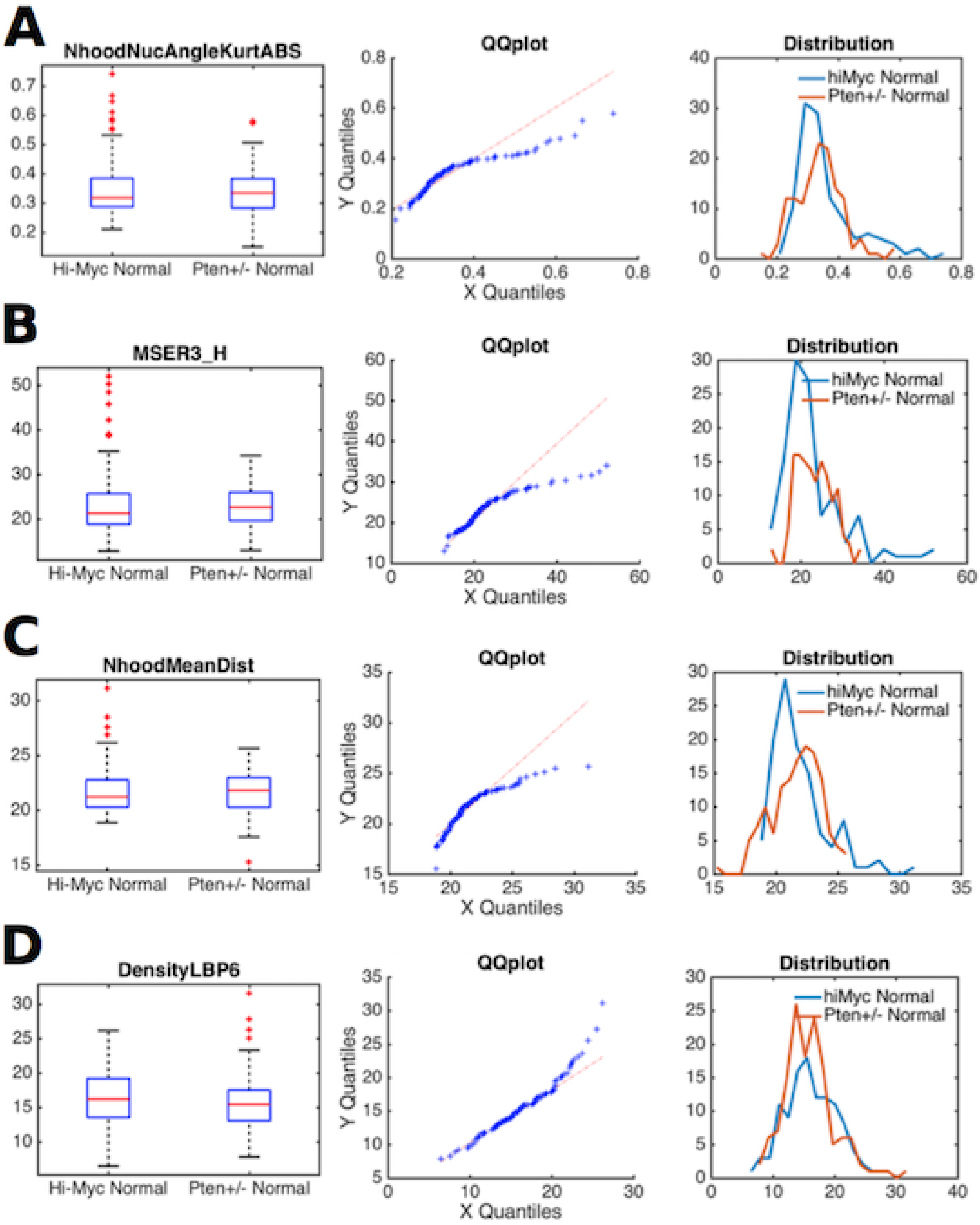

Supplementary Figure 4. Four example features shown in (**A**), (**B**), (**C**), and (**D**) that did not show statistically significant difference between two control groups (normal epithelium of *Pten+/-* and Hi-Myc model mouse prostates). The figures present the feature values as boxplots, QQ-plots, and distributions for each feature.

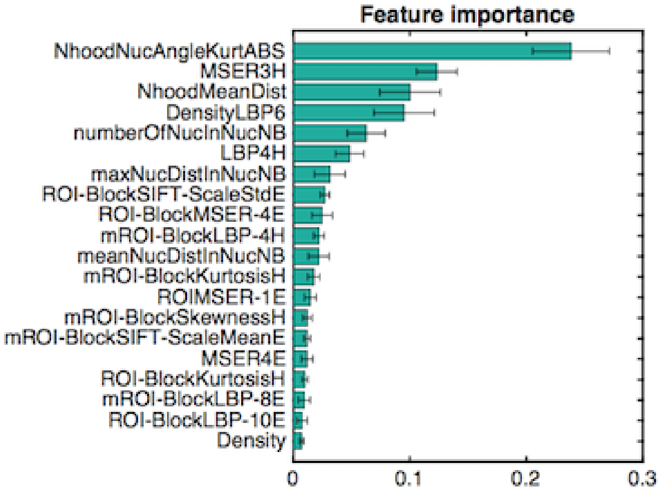

Supplementary Figure 5. The average feature importances and corresponding standard deviations compiled from 582 models trained to distinguish *Pten* heterozygous mPIN, Hi-Myc-induced early neoplasia, and normal epithelium.

Supplementary Table 1. List of extracted features, their description, and the specific neighborhood that the feature is extracted from. All these features were used when assessing spatial variation between mouse prostate lobes, and in distinguishing between *Pten* heterozygous mPIN from normal epithelium.

Feature name	Feature description	Neighborhood	Number of features
Contrast_H, Contrast_E, Correlation_H, Correlation_E, Energy_H, Energy_E, Homogeneity_H, Homogeneity_E	Properties of gray-level co-occurrence matrix from hematoxylin and eosin channel	Bounding box of one segmented nucleus, -> mean value of all nuclei in the ROI area	8
NucAreaSkewness_H, NucAreaSkewness_E, NucAreaKurtosis_H, NucAreaKurtosis_E	Measures of the histogram asymmetry and peakedness from hematoxylin and eosin channel	Bounding box of one segmented nucleus, -> mean value of all nuclei in the ROI area	4
NhoodContrast_H, NhoodContrast_E, NhoodCorrelation_H, NhoodCorrelation_E, NhoodEnergy_H, NhoodEnergy_E, NhoodHomogeneity_H, NhoodHomogeneity_E	Properties of gray-level co-occurrence matrix from hematoxylin and eosin channel	35x35 block around each nucleus, -> mean value of all nuclei in the ROI area	8
NhoodSkewness_H, NhoodSkewness_E, NhoodKurtosis_H, NhoodKurtosis_E	Measures of the histogram asymmetry and peakedness from hematoxylin and eosin channel	35x35 block around each nucleus, -> mean value of all nuclei in the ROI area	4
mROI-BlockContrast_H, mROI-BlockContrast_E, mROI-BlockCorrelation_H, mROI-BlockCorrelation_E, mROI-BlockEnergy_H, mROI-BlockEnergy_E, mROI-BlockHomogeneity_H, mROI-BlockHomogeneity_E	Properties of gray-level co-occurrence matrix from hematoxylin and eosin channel	Masked 50x50 block from ROI area, 50% or more of the block from the ROI area, -> mean value of all blocks in the ROI area	8
mROI-BlockSkewness_H, mROI-BlockSkewness_E, mROI-BlockKurtosis_H, mROI-BlockKurtosis_E	Measures of the histogram asymmetry and peakedness from hematoxylin and eosin channel	Masked 50x50 block from ROI area, 50% or more of the block from the ROI area, -> mean value of all blocks in the ROI area	4
ROI-BlockContrast_H, ROI-BlockContrast_E, ROI-BlockCorrelation_H, ROI-BlockCorrelation_E, ROI-BlockEnergy_H, ROI-BlockEnergy_E, ROI-BlockHomogeneity_H, ROI-BlockHomogeneity_E	Properties of gray-level co-occurrence matrix from hematoxylin and eosin channel	50x50 block from bounding box of the ROI area, -> mean value of all blocks in the image	8
ROI-BlockSkewness_H, ROI-BlockSkewness_E, ROI-BlockKurtosis_H, ROI-BlockKurtosis_E	Measures of the histogram asymmetry and peakedness from hematoxylin and eosin channel	50x50 block from bounding box of the ROI area, -> mean value of all blocks in the image	4
ROISkewness_H, ROISkewness_E, ROIKurtosis_H, ROIKurtosis_E	Measures of the histogram asymmetry and peakedness from hematoxylin and eosin channel	All the pixels from the ROI area	4
ROIintensityVar_H, ROIintensityVar_E, ROIintensitySTD_H, ROIintensitySTD_E, ROIintensityMean_H, ROIintensityMean_E, ROIintensityMax_H, ROIintensityMax_E	Gray level intensity values from hematoxylin and eosin channel	All the pixels from the ROI area	8
meanNucInt_H, meanNucInt_E	Gray level intensity values from hematoxylin and eosin channel	Segmented nuclei area	2
Density	Heat map that represents the density of nuclei, calculated from Gaussian filtered nuclei location map.	One value for each nucleus, -> mean value of all nuclei in the ROI area	1
DensityLBP1-10	Local binary patterns from density heat map. Integer index refers to the LBP histogram bins.	35x35 block around each nucleus, -> mean value of all nuclei in the ROI area	10

ROI-NucDensityPerc10, ROI-NucDensityPerc25, ROI-NucDensityPerc50, ROI-NucDensityPerc75, ROI-NucDensityPerc90	Score below which 10-90% of the heat map gray values are found. Number refers to the calculated percentage.	ROI area	5
NucNhoodDensitySkew, NucNhoodDensityKurt	Measures of the histogram asymmetry and peakedness from density heat map.	35x35 block around each nucleus, -> mean value of all nuclei in the ROI area	2
Distance10neighbours, stdDistance10neighbours, meanDistance10neighbours	Distance measures from one nucleus to its 10 nearest neighbors	One value for each nucleus, -> mean value of all nuclei in the ROI area	3
maxNucDistInNucNB, minNucDistInNucNB, meanNucDistInNucNB, stdNucDistInNucNB	Distance measures between nuclei within neighborhood	100x100 block around each nucleus, -> mean value of all nuclei in the ROI area	4
numberOfNucInNucNB	Number of nuclei within the block	100x100 block around each nucleus, -> mean value of all nuclei in the ROI area	1
NhoodMaxDist, NhoodMeanDist, NhoodStdDist	Distance measures of all nuclei within the block	100x100 block around each nucleus, -> mean value of all nuclei in the ROI area	3
NhoodNucAngleVar, NhoodNucAngleStd, NhoodNucAngleStd0 NhoodNucAngleSkew, NhoodNucAngleSkewABS, NhoodNucAngleSkew0, NhoodNucAngleKurt, NhoodNucAngleKurtABS, NhoodNucAngleKurt0	Circular statistics of angles between center nuclei and other nuclei within the block	100x100 block around each nucleus, -> mean value of all nuclei in the ROI area	9
nucPerLesionSize	Number of nuclei with respect to ROI size	ROI area	1
meanNucSize	Mean area of segmented nucleus within the ROI	Segmented nuclei	1
meanNucDist, stdNucDist, minNucDist	Distance measures between all nuclei within the ROI area	ROI area	3
SIFT-ScaleMean_H, SIFT-ScaleMean_E, SIFT-ScaleStd_H, SIFT-ScaleStd_E, SIFT-NroOfFrames_H, SIFT-NroOfFrames_E	Measures of scale-invariant feature transform from hematoxylin and eosin channel	35x35 block around each nucleus, -> mean value of all nuclei in the ROI area	6
LBP1-10_H, LBP1-10_E	Local binary patterns from hematoxylin and eosin channel. Integer index refers to the LBP histogram bins.	35x35 block around each nucleus, -> mean value of all nuclei in the ROI area	20
MSER1-4_H, MSER1-4_E	Measures of maximally stable extremal regions: mean of ellipse properties (1-3) and number of regions (4)	35x35 block around each nucleus, -> mean value of all nuclei in the ROI area	8
mROI-BlockSIFT-ScaleMean_H, mROI-BlockSIFT-ScaleMean_E, mROI-BlockSIFT-ScaleStd_H, mROI-BlockSIFT-ScaleStd_E, mROI-BlockSIFT-NroOfFrames_H, mROI-BlockSIFT-NroOfFrames_E	Measures of scale-invariant feature transform from hematoxylin and eosin channel	Masked 50x50 block from ROI area, 50% or more of the block from the ROI area, -> mean value of all blocks in the ROI area	6
mROI-BlockLBP-1-10_H, mROI-BlockLBP-1-10_E	Local binary patterns from hematoxylin and eosin channel. Integer index refers to the LBP histogram bins.	Masked 50x50 block from ROI area, 50% or more of the block from the ROI area, -> mean value of all blocks in the ROI area	20

mROI-BlockMSER-1-4_H, mROI-BlockMSER-1-4_E	Measures of maximally stable extremal regions: mean of ellipse properties and number of regions	Masked 50x50 block from ROI area, 50% or more of the block from the ROI area, -> mean value of all blocks in the ROI area	8
ROI-BlockSIFT-ScaleMean_H, ROI-BlockSIFT-ScaleMean_E, ROI-BlockSIFT-ScaleStd_H, ROI-BlockSIFT-ScaleStd_E, ROI-BlockSIFT-NroOfFrames_H, ROI-BlockSIFT-NroOfFrames_E	Measures of scale-invariant feature transform from hematoxylin and eosin channel	50x50 block from bounding box of the ROI area, -> mean value of all blocks in the image	6
ROI-BlockLBP-1-10_H, ROI-BlockLBP-1-10_E	Local binary patterns from hematoxylin and eosin channel. Integer index refers to the LBP histogram bins.	50x50 block from bounding box of the ROI area, -> mean value of all blocks in the image	20
ROI-BlockMSER-1-4_H, ROI-BlockMSER-1-4_E	Measures of maximally stable extremal regions: mean of ellipse properties (1-3) and number of regions (4)	50x50 block from bounding box of the ROI area, -> mean value of all blocks in the image	8
ROISIFT-ScaleMean_H, ROISIFT-ScaleMean_E, ROISIFT-ScaleStd_H, ROISIFT-ScaleStd_E, ROISIFT-NroOfFrames_H, ROISIFT-NroOfFrames_E	Measures of scale-invariant feature transform from hematoxylin and eosin channel	Bounding box of the ROI area	6
ROILBP-1-10_H, ROILBP-1-10_E	Local binary patterns from hematoxylin and eosin channel. Integer index refers to the LBP histogram bins.	Bounding box of the ROI area	20
ROIMSER-1-4_H, ROIMSER-1-4_E	Measures of maximally stable extremal regions: mean of ellipse properties (1-3) and number of regions (4)	Bounding box of the ROI area	8
**Total number of features**			**241**

Supplementary Table 2. List of features selected based on showing no statistically significant difference (significance threshold $\alpha = 0.05$) between the normal samples from *Pten+/-* and Hi-Myc populations in two-sample Kolmogorov-Smirnov test, their description, and the specific neighborhood that the feature is extracted from. These features were used when distinguishing between Pten heterozygous mPIN, Hi-Myc-induced early neoplasia, and normal epithelium.

Feature name	Feature description	Neighborhood	Number of features
Contrast_H, Homogeneity_H	Properties of gray-level co-occurrence matrix from hematoxylin channel	Bounding box of one segmented nucleus, -> mean value of all nuclei in the ROI area	2
NhoodCorrelation_E,<br>NhoodEnergy_E	Properties of gray-level co-occurrence matrix from eosin channel	35x35 block around each nucleus, -> mean value of all nuclei in the ROI area	2
LBP_H (3 pcs),<br>LBP_E (2 pcs)	Local binary patterns from hematoxylin and eosin channel	35x35 block around each nucleus, -> mean value of all nuclei in the ROI area	5
MSER3_H,<br>MSER4_E	Measures of maximally stable extremal regions from hematoxylin and eosin channel: mean of ellipse property 3 and number of regions	35x35 block around each nucleus, -> mean value of all nuclei in the ROI area	2
NhoodNucAngleVar,<br>NhoodNucAngleStd,<br>NhoodNucAngleStd0<br>NhoodNucAngleSkew,<br>NhoodNucAngleKurtABS,<br>NhoodNucAngleKurt0	Circular statistics of angles between center nuclei and other nuclei within the block	100x100 block around each nucleus, -> mean value of all nuclei in the ROI area	6
maxNucDistInNucNB,<br>meanNucDistInNucNB	Distance measures between nuclei within neighborhood	100x100 block around each nucleus, -> mean value of all nuclei in the ROI area	2
numberOfNucInNucNB	Number of nuclei within the block	100x100 block around each nucleus, -> mean value of all nuclei in the ROI area	1
NhoodMeanDist	Distance measure of all nuclei within the block	100x100 block around each nucleus, -> mean value of all nuclei in the ROI area	1
minNucDist	Distance measure between all nuclei within the ROI area	ROI area	1
Density	Heat map that represents the density of nuclei, calculated from Gaussian filtered nuclei location map.	One value for each nucleus, -> mean value of all nuclei in the ROI area	1
DensityLBP (7 pcs)	Local binary patterns from density heat map.	35x35 block around each nucleus, -> mean value of all nuclei in the ROI area	7
mROI-BlockContrast_H,<br>mROI-BlockEnergy_H,<br>mROI-BlockHomogeneity_H	Properties of gray-level co-occurrence matrix from hematoxylin channel	Masked 50x50 block from ROI area, 50% or more of the block from the ROI area, -> mean value of all blocks in the ROI area	3
mROI-BlockSkewness_H,<br>mROI-BlockKurtosis_H	Measures of the histogram asymmetry and peakedness from hematoxylin channel	Masked 50x50 block from ROI area, 50% or more of the block from the ROI area, -> mean value of all blocks in the ROI area	2
mROI-BlockLBP_H (1 pcs),<br>mROI-BlockLBP_E (5 pcs)	Local binary patterns from hematoxylin and eosin channel	Masked 50x50 block from ROI area, 50% or more of the block from the ROI area, -> mean value of all blocks in the ROI area	6
mROI-BlockSIFT-ScaleMean_E	Measures of scale-invariant feature transform from eosin channel: scale mean	Masked 50x50 block from ROI area, 50% or more of the block from the ROI area, -> mean value of all blocks in the ROI area	1
mROI-BlockMSER-1_E	Measures of maximally stable extremal regions: mean of ellipse property 1	Masked 50x50 block from ROI area, 50% or more of the block from the ROI area, -> mean value of all blocks in the ROI area	1

ROI-BlockContrast_H,<br>ROI-BlockHomogeneity_H	Properties of gray-level co-occurrence matrix from hematoxylin channel	50x50 block from bounding box of the ROI area, -> mean value of all blocks in the image	2
ROI-BlockKurtosis_H	Measure of the histogram peakedness from hematoxylin channel	50x50 block from bounding box of the ROI area, -> mean value of all blocks in the image	1
ROI-BlockLBP_H (2 pcs),<br>ROI-BlockLBP_E (3 pcs)	Local binary patterns from hematoxylin and eosin channel	50x50 block from bounding box of the ROI area, -> mean value of all blocks in the image	5
ROI-BlockSIFT-NroOfFrames_H,<br>ROI-BlockSIFT-ScaleStd_E	Measures of scale-invariant feature transform from hematoxylin and eosin channel: number of frames and standard deviation of scales	50x50 block from bounding box of the ROI area, -> mean value of all blocks in the image	2
ROI-BlockMSER-1_E,<br>ROI-BlockMSER-4_E	Measures of maximally stable extremal regions from eosin channel: mean of ellipse property 1 and number of regions	50x50 block from bounding box of the ROI area, -> mean value of all blocks in the image	2
ROIintensityMean_H,<br>ROIintensityMax_H	Gray level intensity values from hematoxylin and eosin channel	All the pixels from the ROI area	2
ROISIFT-ScaleStd_H	Measures of scale-invariant feature transform from hematoxylin channel: standard deviation of scales	Bounding box of the ROI area	1
ROIMSER-1_E	Measure of maximally stable extremal regions from eosin channel: number of regions	Bounding box of the ROI area	1
**Total number of features**			**59**
